# Optimisation of *b*-values for the accurate estimation of the apparent diffusion coefficient (ADC) in whole-body diffusion-weighted MRI in patients with metastatic melanoma

**DOI:** 10.1007/s00330-022-09088-5

**Published:** 2022-09-28

**Authors:** Annemarie K. Knill, Matthew D. Blackledge, Andra Curcean, James Larkin, Samra Turajlic, Angela Riddell, Dow Mu Koh, Christina Messiou, Jessica M. Winfield

**Affiliations:** 1 The Institute of Cancer Research, London, UK; 2 The Royal Marsden NHS Foundation Trust, London, UK; 3 The Francis Crick Institute, London, UK

**Keywords:** Diffusion magnetic resonance imaging, Apparent diffusion coefficient, Whole-body imaging, Melanoma, Metastasis

## Abstract

**Objective:**

To establish optimised diffusion weightings (‘*b*-values’) for acquisition of whole-body diffusion-weighted MRI (WB-DWI) for estimation of the apparent diffusion coefficient (ADC) in patients with metastatic melanoma (MM). Existing recommendations for WB-DWI have not been optimised for the tumour properties in MM; therefore, evaluation of acquisition parameters is essential before embarking on larger studies.

**Methods:**

Retrospective clinical data and phantom experiments were used. Clinical data comprised 125 lesions from 14 examinations in 11 patients with multifocal MM, imaged before and/or after treatment with immunotherapy at a single institution. ADC estimates from these data were applied to a model to estimate the optimum *b*-value. A large non-diffusing phantom was used to assess eddy current–induced geometric distortion.

**Results:**

Considering all tumour sites from pre- and post-treatment examinations together, metastases exhibited a large range of mean ADC values, [0.67–1.49] × 10^−3^ mm^2^/s, and the optimum high *b*-value (*b*_high_) for ADC estimation was 1100 (10th–90th percentile: 740–1790) s/mm^2^. At higher *b*-values, geometric distortion increased, and longer echo times were required, leading to reduced signal.

**Conclusions:**

Theoretical optimisation gave an optimum *b*_high_ of 1100 (10th–90th percentile: 740–1790) s/mm^2^ for ADC estimation in MM, with the large range of optimum *b*-values reflecting the wide range of ADC values in these tumours. Geometric distortion and minimum echo time increase at higher *b*-values and are not included in the theoretical optimisation; *b*_high_ in the range 750–1100 s/mm^2^ should be adopted to maintain acceptable image quality but performance should be evaluated for a specific scanner.

**Key Points:**

*• Theoretical optimisation gave an optimum high b-value of 1100 (10th–90th percentile: 740–1790) s/mm*^*2*^ *for ADC estimation in metastatic melanoma.*

*• Considering geometric distortion and minimum echo time (TE), a b-value in the range 750–1100 s/mm*^*2*^ *is recommended.*

*• Sites should evaluate the performance of specific scanners to assess the effect of geometric distortion and minimum TE.*

**Supplementary Information:**

The online version contains supplementary material available at 10.1007/s00330-022-09088-5.

## Introduction

Whole-body MRI with diffusion-weighted imaging (WB-DWI) has emerged as a powerful tool for monitoring patients with advanced cancers because it offers combined morphological and quantitative information within a single radiological exam [1, 2]. It has a favourable safety profile, due to the lack of ionising radiation used in other techniques such as CT and PET-CT, and a good diagnostic performance [3, 4]. WB-MRI offers an increased overall specificity and sensitivity in the liver, subcutaneous and intramuscular lesions over CT [5]. Acquiring images with at least two diffusion weightings (‘*b*-values’) enables quantification of the apparent diffusion coefficient (ADC), a surrogate biomarker of tumour cellularity. Inclusion of WB-DWI in imaging guidelines [6–8] for staging and response evaluation in myeloma and metastatic prostate cancer has triggered standardised protocols optimised for those applications [1, 2, 9]. The lack of ionising radiation has also seen WB-DWI evaluated as a screening tool for patients with high risk of developing cancer [10, 11].

WB-DWI has not yet been extensively investigated in metastatic melanoma (MM), and current evidence is therefore limited. An evaluation of the imaging characteristics in these patients is required to determine a suitable protocol before WB-DWI can be used for larger studies in patients with MM, because there is no guarantee that existing protocols will be suitable for imaging MM. However, WB-DWI has great potential for detecting small volume disease in these patients and has already been included in some national guidelines for imaging paediatric patients [12].

Immunotherapy in the form of the immune checkpoint inhibitors ipilimumab, nivolumab and pembrolizumab has improved survival for patients with MM but introduces new challenges when imaging response. Atypical response patterns include disease shrinkage after initial increase in tumour burden, and shrinkage after appearance of new lesions; the reported incidence of this ‘pseudoprogression’ is up to 10% [13]. Potential for highly morbid toxicities [14, 15] and the cost of treatment accentuate the challenges of maintaining patients on a therapy where therapeutic benefit is uncertain for individual patients.

WB-DWI offers potential as a sensitive staging tool and whole-body estimates of tumour ADC may also reflect response to treatment independent of changes in lesion size [16]. However, to date there is insufficient data to allow optimisation of *b*-values for ADC estimation and response assessment of MM.

The aim of this study was to establish optimised *b*-values for the acquisition of WB-DWI for ADC estimation in patients with MM, using retrospective patient data and phantom experiments. It was also necessary to assess the practical implementation and the effects on image quality, including geometric distortion and signal-to-noise ratio (SNR), when acquiring images with optimum *b*-values on clinical MRI scanners.

## Materials and methods

### Clinical studies

A retrospective single-institution study was performed, with the requirement for written informed consent waived by the institution. All patients with an established diagnosis of MM who underwent an MRI examination of the whole body or abdomen and pelvis including diffusion-weighted imaging (DWI) between May 2017 and February 2019 were included. Patients with previously treated and untreated disease were included. None of the scans were excluded. Images were acquired on either a 1.5-T or 3-T scanner (MAGNETOM Aera, Avanto and Skyra, Siemens Healthcare) (Table 1).

**Table 1:** MRI acquisition parameters

		Abdo/pelvis		
Scanning parameters	WB-DWI	A	B	C
B_0_ field strength (T)	1.5	3	1.5	1.5
Scanner model	Aera	Skyra	Aera	Avanto
Sequence	SE-EPI	SE-EPI	SE-EPI	SE-EPI
Orientation	Axial	Axial	Axial	Axial
*b*-values (s/mm^2^)	50, 600, 900	50, 600, 900	50, 600, 900	50, 600, 900, 1050
Diffusion gradient scheme	Monopolar	Bipolar	Monopolar	Bipolar
Diffusion encoding scheme ^a^	Four-scan trace	Three-scan trace	Four-scan trace	Three-scan trace
Echo time (ms)	64	70	61	71
Repetition time (ms)	6150	10,900	10,200	8000
Slices per station/stations	40/6	50/2	50/2	50/3
Slice thickness (mm)	5	5.5	5 or 6	5
Slice gap (mm)	0	0	0	0
Field of view (mm)	354 × 439 or 346 × 430	313 × 380	446 × 439	420 × 341
Reconstructed matrix (mm × mm)	268 × 216	320 × 264	272 × 268	256 × 208
Acquisition matrix (mm × mm)	134 × 108	160 × 132	136 × 134	128 × 104
Fat suppression	STIR	SPAIR	SPAIR	SPAIR
Breathing instructions	Free breathing	Free breathing	Free breathing	Free breathing
Number of signal averages per *b*-value	2, 2, 4	1, 3, 3	2, 2, 4	3, 3, 3, 3
**Patients**	**5**	**3**	**2**	**1**

^a^Three-scan trace employs three mutually orthogonal diffusion gradient directions, which are not aligned with the cardinal axes of the scanner; four-scan trace is an implementation of the tetrahedral diffusion encoding scheme [30]

All scanners made by Siemens Healthcare. *SE-EPI*, Spin Echo - Echo Planar imaging; *STIR*, short T_1_ inversion recovery; *SPAIR*, spectral adiabatic inversion recovery

**Fig. 1:**
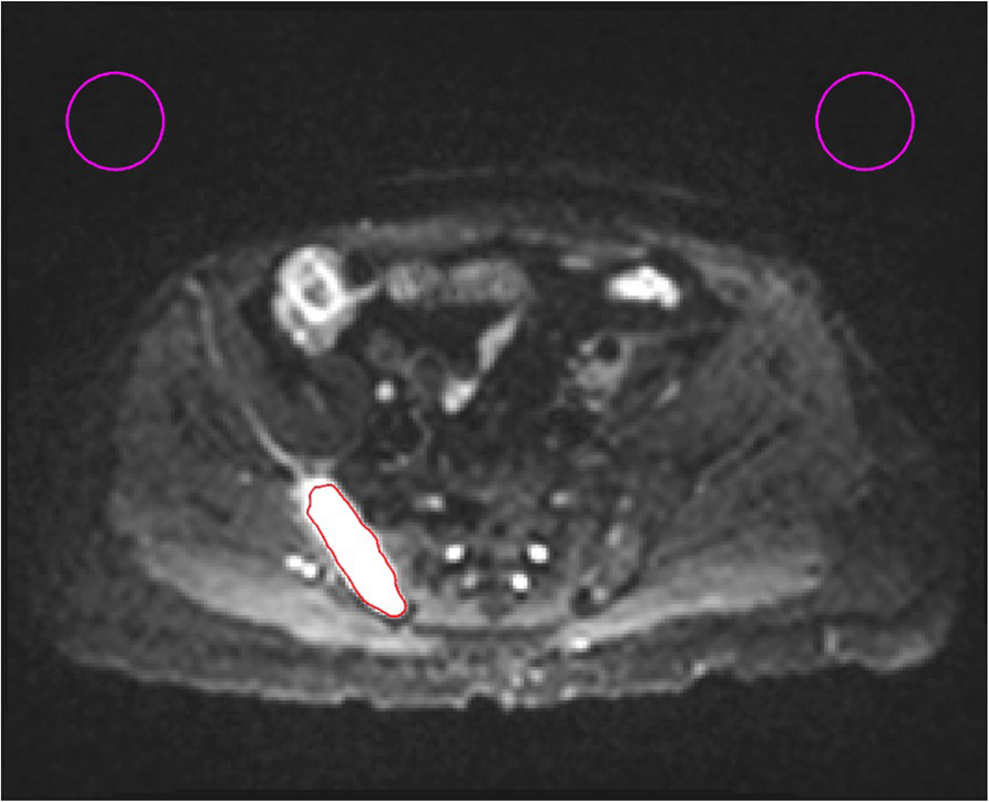
An example of the ROIs drawn (pink) on DWI *b* = 50 s/mm^2^ image to estimate the noise. The lesion is delineated in red. The number of pixels in the ROIs was kept constant across the different matrix sizes used

For each patient, regions of interest (ROIs) were drawn around all tumours larger than 1 cm in size on *b* = 50 s/mm^2^ images on all tumour containing image slices and checked by a radiologist with > 1 year of experience in WB-DWI (Fig. 1). ROIs were drawn using Horos (horosproject.org), and morphological sequences were used as reference. The minimum tumour diameter of 1 cm was chosen for ROIs to avoid partial volume effects. ADCs were estimated using a mono-exponential decay model fitted to the logarithm of the DWI signal at all *b*-values using a least squares fit (SciPy [17]). The mean ADC of all fitted voxels in the ROIs was estimated for each tumour.

#### Image and data analysis

Using error propagation and the definition of the ADC, Bito et al proposed a model for the standard deviation in the ADC when images are acquired with at least two *b*-values,

1 $$ \mathrm{SD}(D)={D}_0\sigma \frac{\sqrt{\sum_{n=1}^N{\left(N{x}_n-{\sum}_{i=1}^N{x}_i\right)}^2\exp \left(2{x}_n\right)}}{N{\sum}_{n=1}^N{x_n}^2-{\left({\sum}_{n=1}^N{x}_n\right)}^2}, $$

where *b*_low_ is the lowest *b*-value used, *x*_*n*_ = *D*_0_*b* are the scaled gradient factors, *σ* is the standard deviation of the signal in the image acquired with *b*_*low*_, *D*_0_ is the true ADC, and *N* is the number of image acquisitions [18]. This model assumes that signal attenuation follows mono-exponential decay with *b*-value and that noise in the signal is normally distributed with variance *σ*^2^. It provides a general description of the variation in SD(*D*) given any combination of *b*-values; however, optimisation yields a solution requiring only two: *b*_low_ and *b*_high_. From Eq. 1, the optimum ratio, *R*, of the number of image acquisitions at the low to high *b*-value is 1:3 [18]. The optimum value of *b*_*low*_ is 0 s/mm^2^; however, many authors choose 50 s/mm^2^ to reduce the influence of perfusion [18, 19].

In each of the *b* = 50 s/mm^2^ images in which the tumours were delineated, two identical circular ROIs were drawn in a region of background, away from signal, ghosts and unsuppressed fat (Fig. 1). The value of *σ* was estimated by taking the standard deviation of all pixels in the background ROIs. The mean tumour ADCs of the voxels in the delineated lesions were calculated as an estimate of the population ADC, *D*_0_, in each sub-group of the lesions, split by tissue type and scanning time point, and overall.

The optimum high *b*-value (*b*_opt_) was estimated computationally as the value of *b*_high_ which minimised SD(*D*) calculated for *b*_high_ in the range 400–2500 s/mm^2^ with *b*_low_ = 50 s/mm^2^, *N* = 24 and *R* = 1:3. The value of *b*_opt_ was estimated using *σ* and *D*_0_ calculated from each sub-group of the lesions and overall. The validity of the model was confirmed experimentally by comparing the measured standard deviation of the ADC of a uniform phantom to the prediction made using the model (as detailed in the Supplementary Materials).

### Phantom studies

#### Variation in echo time with *b*-value

The minimum achievable echo time (TE) and corresponding repetition time (TR) were noted for high *b*-values in the range 700–1800 s/mm^2^ (lowest *b*-value = 50 s/mm^2^, FOV = 500 × 500 mm^2^, and matrix size = 124 × 138, MAGNETOM Aera Scanner, Siemens Healthcare).

#### Evaluation of *b*-value dependence on geometric distortion

**Fig. 2:**
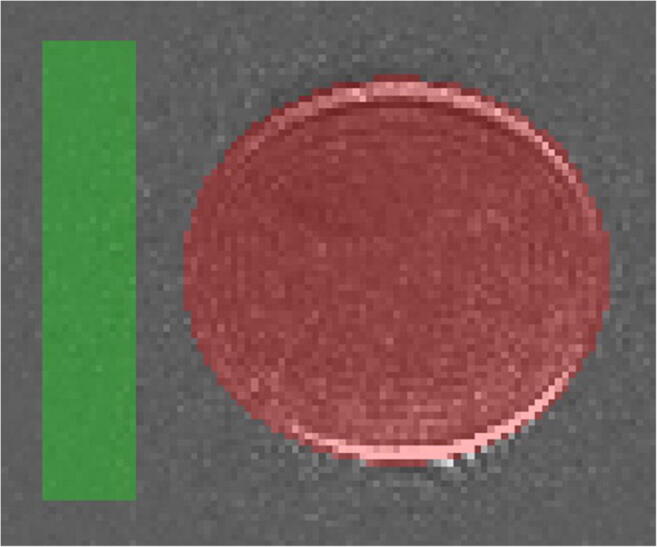
An example of the ROIs drawn on the DWI subtraction images to calculate the semi-quantitative distortion index using a PDMS phantom. ROIs are drawn in the signal region (376 cm^2^, red) and in a region of noise (130 cm^2^, green) on a single central slice. Axial DWI was acquired with TR = 11,200 ms, TE = 80 ms and *b*-values 0, 900, 1050, 1150 and 1250 s/mm^2^ using multi-directional diffusion weightings, bipolar gradients with 12 diffusion encoding directions

Geometric distortion was assessed using a large phantom containing polydimethylsiloxane (PDMS) [20, 21]. Axial DWI was acquired with TR = 11,200 ms, TE = 80 ms and *b*-values 0, 900, 1050, 1150 and 1250 s/mm^2^ using multi-directional diffusion weightings and bipolar gradients with 12 diffusion encoding directions. ROIs were drawn on subtraction images (the signal intensity of the *b* = 0 s/mm^2^ minus the high *b*-value image) around the phantom and a region of noise (Fig. 2). A semi-quantitative distortion index (DI) was calculated by counting the number of pixels in the phantom ROI which had a value 3 times greater than the standard deviation of the pixels in the noise ROI and dividing by a factor of 1000.

## Results

### Clinical studies

Eleven patients with MM, 6 males and 5 females with a mean age of 58 years (range 22–73 years), underwent MRI investigations. These included 5 previously treated patients (3 males, 2 females) and 6 untreated patients (3 males, 3 females). None of the patients had previous or coexisting malignancies. Three patients had baseline and post-immunotherapy scans; 4 patients had baseline scans only; 4 patients had a single post-treatment scan. Of the patients with post-treatment scans, 3 were treated with pembrolizumab (1 reported as response, 1 stable disease, 1 progression), 1 with ipilimumab (progression) and 3 with a combination of ipilimumab and nivolumab (1 reported as response, 1 stable disease, 1 progression, later confirmed as pseudoprogression). The mean time between the most recent cycle of immunotherapy and the post-treatment scan was 92 days (range: 7–250 days). Eight patients were scanned at 1.5 T and three patients scanned at 3 T. Of these patients, five were scanned with whole-body coverage and six covering the abdomen and pelvis. The volume and distribution of disease are outlined in Table 2. A total of 125 lesions were analysed in this study, with the most common locations of metastasis being the bone, liver and lymph nodes.

**Table 2:** Distribution and volume of the analysed lesions

	No. of patients			No. of Tumours			Median lesion volume (cm3)		
Location	Pre Rx	Post Rx	Overall	Pre Rx	Post Rx	Overall	Pre Rx	Post Rx	Overall
Bone	3	4	5	29	19	48	22.9 (7.81–248)	16.0 ((8.07–471)	19.7 (7.81–471)
Liver	4	3	4	21	26	47	26.6 (11.1–53,900)	156 (16.1–71,200)	57.3 (16.1–71,200)
Lymph nodes	4	3	4	5	5	10	121 (8.96–929)	183 (17.9–2130)	126 (8.96–2130)
Bowel/Peritoneum	2	4	6	2	5	7	609 (18.6–1200)	59.7 (13.6–205)	60 (13.6–1200)
Lung	2	1	2	3	1	4	109 (25.2–4060)	5670	2080 (25.2–5670)
Kidney	0	2	2		2	2		16.0 (7.93–24.0)	16.0 (7.93–24.0)
Subcutaneous	1	1	1	1	1	2	31.4	22.8	27.1 (22.8–31.4)
Muscle	1	2	2	0	2	2		1250 (310–2200)	1250 (310–2200)
Other	2	0	2	2	0	2	24.5 (17.6–31.5)		24.5 (17.6–31.5)
Adrenal		1	1	0	1	1		41	41
**Overall**	**7**	**7**	**11**	**63**	**62**	**125**	**28.3 (7.81–53,900)**	**64.1 (7.93–71,200)**	**40.6 (7.81–71,200)**

Values in brackets show the range in volume. *Rx*, treatment

The mean ADC estimates from sites of disease at different anatomical locations are summarised in Table 3. The lowest mean ADC was calculated for the group ‘other’ (two presacral nodules) 0.67 × 10^−3^ mm^2^/s, and the highest mean ADC was in intramuscular deposits, 1.49 × 10^−3^ mm^2^/s.

**Table 3:** Mean and 10th and 90th percentile of the ADCs

	Mean ADC (10−^3^ mm^2^/s)		
Location	Pre Rx	Post Rx	Overall
Bone	0.91 (0.50–1.48)	1.33 (0.66–1.86)	1.11 (0.56–1.74)
Liver	1.13 (0.68–1.82)	1.20 (0.77–1.80)	1.18 (0.74–1.81)
Lymph nodes	1.42 (0.81–2.26)	1.29 (0.82–1.84)	1.32 (0.82–1.99)
Bowel/Peritoneum	0.91 (0.67–1.23)	0.85 (0.43–1.24)	0.90 (0.65–1.23)
Lung	1.11 (0.52–1.87)	1.08 (0.59–1.84)	1.10 (0.56–1.85)
Kidney		1.38 (0.99–1.79)	1.38 (0.99–1.79)
Subcutaneous	1.05 (0.65–1.66)	0.96 (0.73–1.31)	1.01 (0.69–1.45)
Muscle		1.49 (1.20–1.77)	1.49 (1.20–1.77)
Other	0.67 (0.48–0.86)		0.67 (0.48–0.86)
Adrenal		1.00 (0.50–1.54)	1.00 (0.50–1.54)
**Overall**	**1.12 (0.65–1.81)**	**1.20 (0.75–1.80)**	**1.18 (0.72–1.81)**

10th and 90th percentile in brackets. The group classified as ‘other’ includes two presacral nodules. *Rx*, treatment

**Fig. 3:**
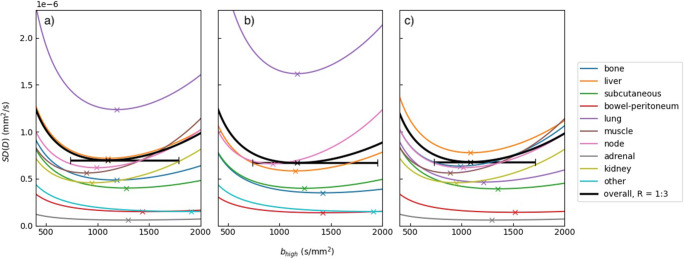
From left to right, the standard deviation in the ADC plotted against *b*_high_ for all timepoints, pre-treatment lesions only and post-treatment lesions only, using a ratio, *R*, equal to the optimum value of 1:3 and *b*_low_ = 50 s/mm^2^. The retrospective data is used to estimate the true ADC and *D*_0_ and the number of image acquisitions, *N*, is 24. The minima of the curves (×) correspond to the predicted optimum *b*-value, *b*_opt_, for each tumour type. The error bars on the minima of the curves represent the values of *b*_opt_ calculated using the 10th and 90th percentile of the retrospective ADC estimates. (On clinical MRI scanners, *b*-values are often specified in increments of 50 s/mm^2^)

The value of *b*_opt_ is estimated to be 1100 (10th and 90th percentile: 740–1790) s/mm^2^ considering lesions at all timepoints and locations, as shown in Fig. 3.

### Phantom studies

#### Variation in TE with *b*-value

For the sequence described, at *b* = 700 s/mm^2^ the minimum TE was 68 ms with TR = 6270 ms, and at *b* = 1800 s/mm^2^ the minimum TE was 81 ms with TR = 6372 ms. The minimum TE increases with the *b*-value in the evaluated range (700–1800 s/mm^2^).

#### Evaluation of the *b*-value dependence of distortion

**Fig. 4:**
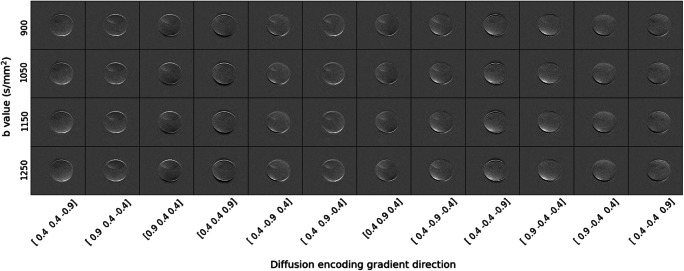
Subtraction images, calculated by subtracting the signal intensity in the image with the given *b*-value and diffusion encoding direction from the image acquired at *b* = 0 s/mm^2^. All images have the same window width and window level

**Fig. 5:**
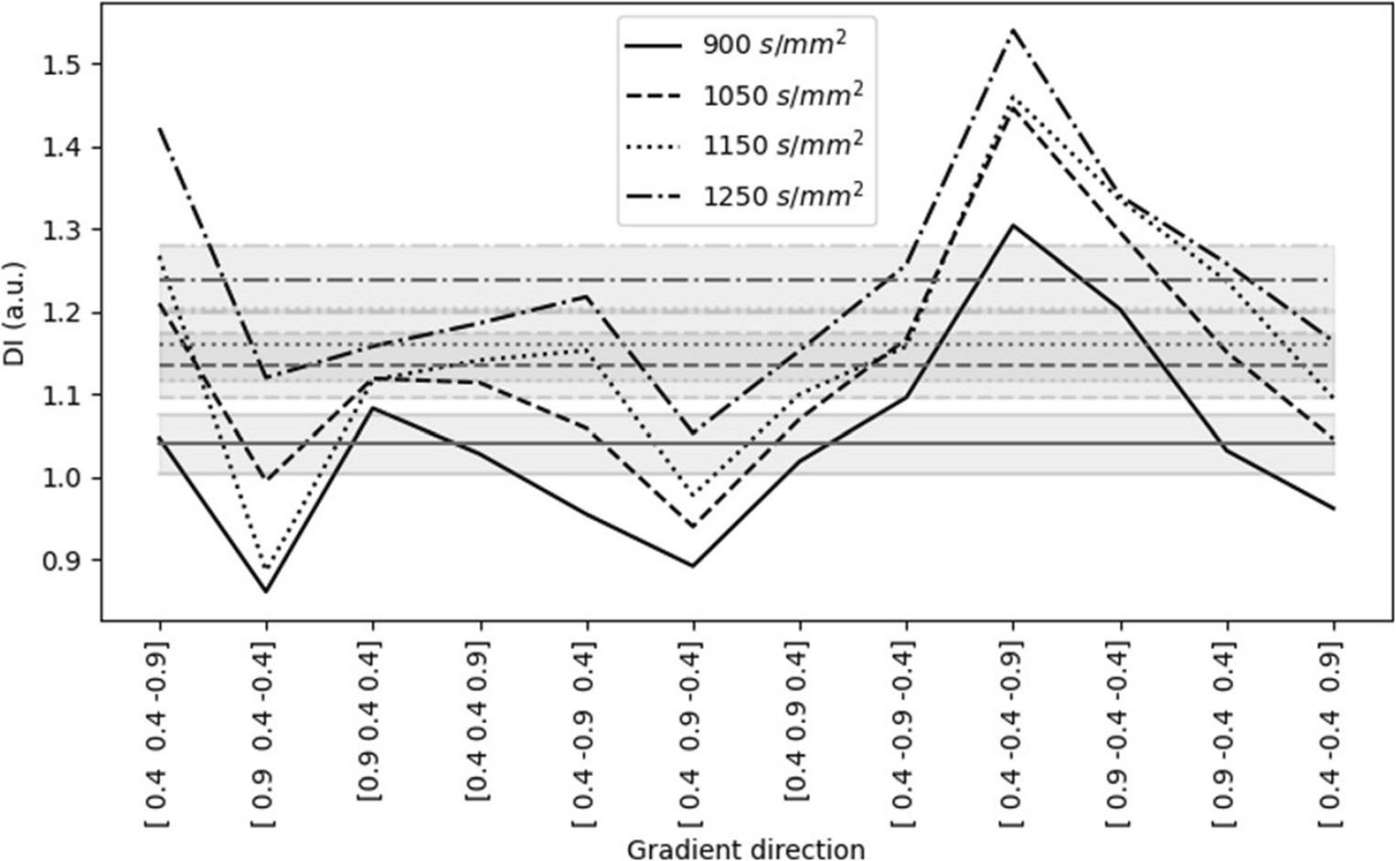
The semi-quantitative distortion index calculated from DWI of a PDMS phantom when images are acquired with different gradient encoding directions, *b*_low_ = 0 s/mm^2^ and *b*_high_ as shown in the figure legend. The horizontal grey lines represent the mean values across all diffusion encoding directions at the given *b*-value and the shaded regions show the corresponding standard error on these means

The subtraction images calculated using the PDMS phantom are shown in Fig. 4. Figure 5 shows the results of the analysis using two ROIs specified in Fig. 2. While there is a large amount of variation in the semi-quantitative DI at different encoding directions, the mean of the DI across all diffusion encoding directions for a given *b*-value is noticeably higher at *b*-values = 1050, 1150, and 1250 s/mm^2^ than at *b* = 900 s/mm^2^.

## Discussion

Protocol recommendations for WB-DWI in myeloma and metastatic prostate cancer recommend high *b*-values of 800–1000 s/mm^2^ and 800–900 s/mm^2^ respectively [1, 2]; however, a recommendation of the choice of *b*-values for use in MM has not yet been established. To achieve this, an estimation of the range of ADC values in these patients was necessary.

This retrospective study of patients with metastatic melanoma demonstrated that a large range of mean ADCs exists across disease sites and pre- and post-treatment: [0.67–1.49] × 10^−3^ mm^2^/s and the overall mean ADC values calculated were comparable to the reported ADC of other tumour types, such as myeloma [22–24] and metastatic prostate cancer [25].

From this analysis, the theoretical optimum *b*-value for the estimation of ADC values in MM was 1100 (10th and 90th percentile: 740–1790) s/mm^2^. The large range of mean ADCs in this disease type is reflected in the difference between the values of the 10th and 90th percentile of *b*_opt_. Lesions with high ADC and short T_2_ values will have very low signal at high *b*-values above 1100 s/mm^2^, leading to noise bias in ADC estimation. Hence, a *b*-value in the range 750–1100 s/mm^2^ is an appropriate compromise as it reduces the noise bias in these low signal regions, despite not being the optimum *b*-value for some lesions.

The *b*-value and the echo time (TE) of a DWI sequence are interrelated, so when the *b*-value is increased the TE also increases [26]. This influences the T_2_ weighting of the images. However, the T_2_ dependence of SD(*D*) is not examined in Eq. 1, and in order to apply any model which accounts for the dependence on T_2_ (for example, Saritas et al [27]), the characteristic values of the T_2_ relaxation time for melanoma tumours would be needed. Such information has not yet been published. The approximate signal loss can be estimated from the minimum values of TE, an estimate of T_2_ for melanoma (41 ms, from a pre-clinical model [28]), and by considering only T_2_ decay. This suggests that there would be ~27% lower signal in images acquired at TE = 81 ms (the minimum TE at *b* = 1800 s/mm^2^, for the sequence investigated in this study) compared with images acquired at TE = 68 ms (the minimum TE at *b* = 700 s/mm^2^).

It is also important to consider the increase in eddy current–induced distortion with *b*-value. Increased distortion at high *b*-values will impact the accuracy of the pixelwise ADC calculation because pixels are no longer aligned on the images acquired at different *b*-values [20]. The results from the PDMS phantom demonstrated mean distortion across all diffusion encoding directions was noticeably higher when acquiring images with *b*-values 1050, 1150 and 1250 s/mm^2^ compared to acquisition with *b* = 900 s/mm^2^.

Reduction in signal and increase in distortion at higher *b*-values can result in impaired image quality and degraded ADC estimates. Distortion and achievable TE effects are scanner dependent; therefore, centres may choose to assess the individual performance of their scanners before deciding on the high *b*-value for their protocol.

Most clinically adopted DWI protocols will also include a third *b*-value midway between the minimum and maximum values, usually around 600 s/mm^2^ [1, 2]. This can be useful for the observation of soft tissues and can also be included in the calculation of the ADC.

The key limitation of this study was the heterogeneity of the retrospective data due to variations in the scanner, field strength and parameters. Whilst these differences may affect properties such as SNR, diffusion times and fat signal, the differences between parameters are relatively small (comparable to the ranges specified in the QIBA liver profile [29]) and reflect the variation in imaging protocols used for different body parts and scanners. Additionally, the ADC is not dependent on the field strength and the TR in all protocols was sufficiently long to allow for T_1_ recovery. Therefore, variation in these parameters is not expected to have a marked impact on the presented results.

Furthermore, the differences in parameters across the protocols reflect the nature of real-world data. This is in some ways an advantage; the recommendations we draw from this cohort have been derived from a mixture of data; therefore, the values of the mean ADC and noise in the images are not biased towards one protocol. The optimisation of the technical aspects of the imaging protocol presented here provides an ideal starting point for the collection of larger data sets in this patient population.

Moreover, with the advent of immunotherapy treatment, the proportion of MM patients who receive systemic treatment is increasing. Therefore, the characterisation of melanoma lesions both pre- and post-treatment is important to ensure the proposed *b*-values are appropriate for this group of patients at all stages of treatment.

In conclusion, the analysis of the retrospective data has demonstrated that there is a large range of mean ADC values which are characteristic of MM. Using only a theoretical model, a high *b*-value of 1100 (10th and 90th percentile: 740–1790) s/mm^2^ with *b*_low_ = 50 s/mm^2^ was determined to be optimal for ADC calculation; however, due to the additional considerations of increased distortion and minimum TE at high *b*-values, we recommend *b*_high_ is chosen in the range 750–1100 s/mm^2^ and individual sites should assess the performance of their scanners before choosing a value for their protocol.

## Supplementary information

ESM 1 (PDF 348 kb)

## Abbreviations

ADC: Apparent diffusion coefficient
DI: Distortion index
DWI: Diffusion-weighted imaging
MM: Metastatic melanoma
PDMS: Polydimethylsiloxane
ROI: Region of interest
Rx: Treatment
SE-EPI: Spin Echo - Echo Planar imaging
SNR: Signal-to-noise ratio
SPAIR: Spectral Attenuated Inversion Recovery
STIR: Short T_1_ inversion recovery
TE: Echo time
TR: Repetition time
WB-DWI: Whole-body diffusion-weighted MRI

## Declarations

### Guarantor

The scientific guarantor of this publication is Christina Messiou.

### Statistics and biometry

No complex statistical methods were necessary for this paper.

### Methodology

• retrospective

• observational

• performed at one institution
