# Supplementary material for: Optimisation of b-values for the accurate estimation of the apparent diffusion coefficient (ADC) in whole-body diffusion-weighted MRI in patients with metastatic melanoma
Source: Eur Radiol. 2022 Sep 28;33(2):863–71. doi: 10.1007/s00330-022-09088-5 (PMC9889461; doi:10.1007/s00330-022-09088-5)
Supplement: Supplementary file 1 — (PDF 348 kb) [file 330_2022_9088_MOESM1_ESM.pdf]

Supplementary Material:

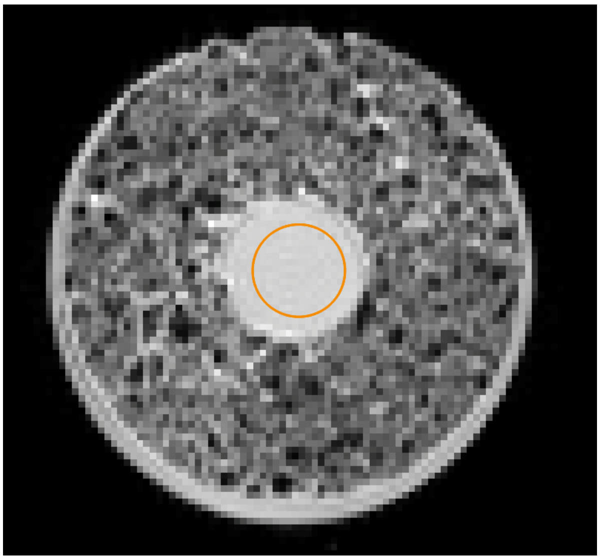

Figure 1: A diffusion weighted image (b=1000 s/mm^2) of a temperature-controlled phantom, containing deionised water surrounded by ice. 159 voxels from 4 slices of the ADC map in the region delineated in orange were used to evaluate the accuracy of a theoretical model describing the variation in the standard deviation of the ADC when acquired with different choices of b-values.

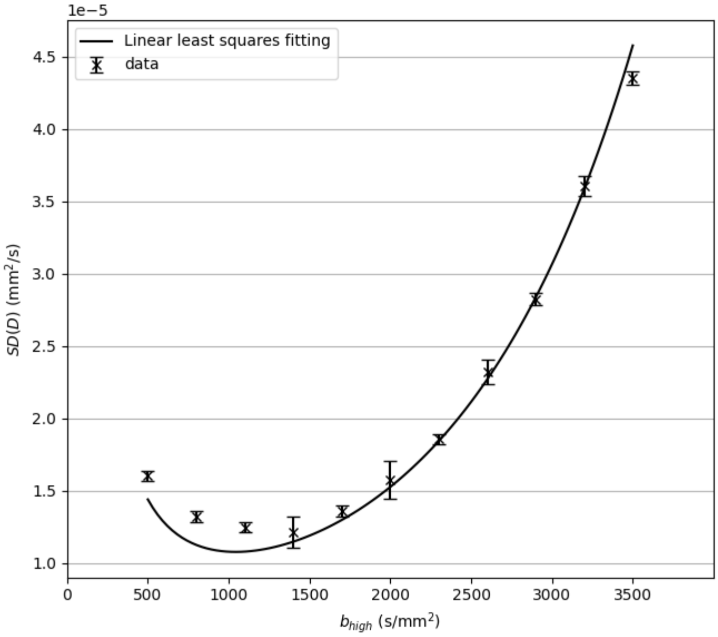

Figure 2: The variation of the standard deviation in the ADC, SD(D), in the VOI (Figure 2) when calculated using DWI acquired with b_{low} = 50 s/mm^2 and b_{high}. The solid line shows a linear least squares fitting to the data using the model described by Equation 1. Error bars are calculated from the standard deviation of a bootstrapped distribution of SD(D) with 1000 iterations.

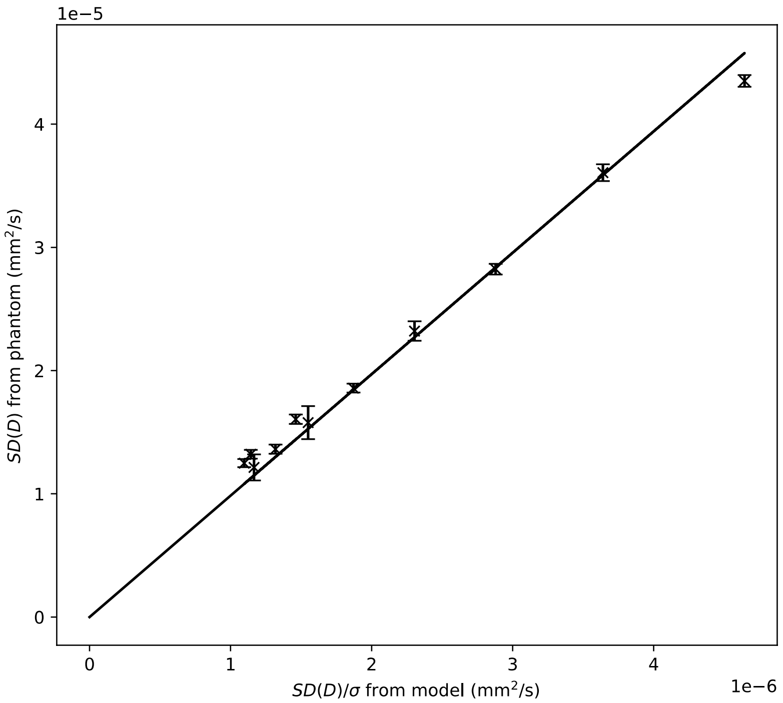

Figure 3: The standard deviation of the ADC, SD(D), in the VOI (Figure 2), plotted against the predicted value of SD(D)/σ calculated using the model described in Equation 1. The solid line shows a least squares fitting to the data with an intercept forced through the origin and gradient = 9.86. Error bars are calculated from the standard deviation of a bootstrapped distribution of SD(D) with 1000 iterations.

Experimental comparison with *b*-value optimisation:

Method:

A temperature-controlled Perspex phantom containing deionised water was used to investigate experimentally the change in the standard deviation of the calculated ADC with the *b*-value, Figure 1. Axial DWI was acquired with TR = 5100 ms, TE = 98 ms, b_{low} = 50 s/mm^2 and b_{high} in the range [500 – 3500] s/mm^2 using multidirectional diffusion weighted (MDDW) gradients with 12 diffusion encoding directions. The pixel-wise ADC maps were calculated using a least squares fitting to the logarithm of the signal intensity in the 24 images acquired at b_{low} and b_{high}.

ROIs were drawn in 4 axial slices; these were combined to form a volume of interest (VOI). The standard deviation in the ADC was found using the ADC estimates from the voxels in the VOI. Bootstrapping was used to estimate the error, where the distribution of ADC values was resampled 1000 times with replacement.

Results:

The standard deviation in the ADC, SD(D), of deionised water in a temperature controlled phantom is plotted on the y axis of Figure 2 and Figure 3. Error bars are calculated from the standard deviation of a bootstrapped distribution of SD(D) with 1000 iterations.

In Figure 2, SD(D) is plotted against the value of b_{high} used to acquire the DWI, where b_{low} = 50 s/mm^2. A least squares fitting was used to fit the model described in Equation 1 to the data and estimate the value of σ, where D_0 was estimated from a mean of the ADC in the VOI in three series acquired with b_{low} = 50 s/mm^2 and b_{high} = 800 s/mm^2.

In Figure 3, SD(D) is plotted against the theoretical estimate of SD(D)/σ calculated using Equation 1, where D_0 was estimated in the same way as described above. The gradient of the

linear fitting with an intercept through the origin is an estimate of the value of σ. The results of this fitting estimate $\sigma = 9.86$ with a goodness of fit statistic $r^2 = 0.986$.

Discussion:

The comparison of the theoretical optimisation to measurements of the SD(D) in a temperature controlled phantom showed that although the model slightly underestimated the value of SD(D) at points acquired with *b*-values less than 1400 s/mm^2, there was good overall agreement with the experimental data.
